# The Smallest Spectral Radius of Graphs with a Given Clique Number

**DOI:** 10.1155/2014/232153

**Published:** 2014-07-13

**Authors:** Jing-Ming Zhang, Ting-Zhu Huang, Ji-Ming Guo

**Affiliations:** ^1^School of Mathematical Sciences, University of Electronic Science and Technology of China, Chengdu, Sichuan 611731, China; ^2^College of Science, China University of Petroleum, Qingdao, Shandong 266580, China; ^3^College of Science, East China University of Science and Technology, Shanghai 200237, China

## Abstract

The first four smallest values of the spectral radius among all connected graphs with maximum clique size *ω* ≥ 2 are obtained.

## 1. Introduction

Let *G* = (*V*(*G*), *E*(*G*)) be a simple connected graph with vertex set *V*(*G*) = {*v*_1_, *v*_2_,…, *v*_*n*_} and edge set *E*(*G*). Its adjacency matrix *A*(*G*) = (*a*_*ij*_) is defined as *n* × *n* matrix (*a*_*ij*_), where *a*_*ij*_ = 1 if *v*_*i*_ is adjacent to *v*_*j*_ and *a*_*ij*_ = 0, otherwise. Denote by *d*(*v*_*i*_) or *d*_*G*_(*v*_*i*_) the degree of the vertex *v*_*i*_. It is well known that *A*(*G*) is a real symmetric matrix. Hence, the eigenvalues of *A*(*G*) can be ordered as

(1) $$\begin{array}{c} \lambda_{1}\left(G\right)\geq \lambda_{2}\left(G\right)\geq \cdots \geq \lambda_{n}\left(G\right), \end{array}$$

respectively. The largest eigenvalue of *A*(*G*) is called the spectral radius of *G*, denoted by *ρ*(*G*). It is easy to see that if *G* is connected, then *A*(*G*) is nonnegative irreducible matrix. By the Perron-Frobenius theory, *ρ*(*G*) has multiplicity one and exists a unique positive unit eigenvector corresponding to *ρ*(*G*). We refer to such an eigenvector corresponding to *ρ*(*G*) as the Perron vector of *G*.

Denote by *P*_*n*_ and *C*_*n*_ the path and the cycle on *n* vertices, respectively. The characteristic polynomial of *A*(*G*) is det⁡(*xI* − *A*(*G*)), which is denoted by Φ(*G*) or Φ(*G*, *x*). Let *X* be an eigenvector of *G* corresponding to *ρ*(*G*). It will be convenient to associate with *X* a labelling of *G* in which vertex *v*_*i*_ is labelled *x*_*i*_ (or *x*_*v*_*i*__). Such labellings are sometimes called “valuation” [1].

**Figure 1:**
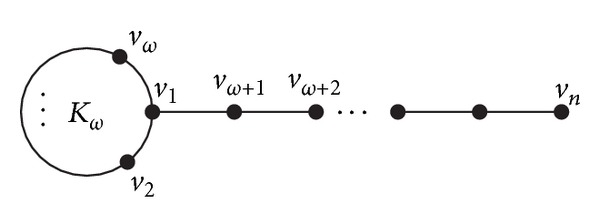
Kite graph *PK*_*n*−*ω*,*ω*_.

The investigation on the spectral radius of graphs is an important topic in the theory of graph spectra. The recent developments on this topic also involve the problem concerning graphs with maximal or minimal spectral radius, signless Laplacian spectral radius, and Laplacian spectral radius, of a given class of graphs, respectively. The spectral radius of a graph plays an important role in modeling virus propagation in networks [2]. It has been shown that the smaller the spectral radius, the larger the robustness of a network against the spread of viruses [3]. In [4], the first three smallest values of the Laplacian spectral radii among all connected graphs with maximum clique size *ω* are given. And, in [5], it is shown that among all connected graphs with maximum clique size *ω* the minimum value of the spectral radius is attained for a kite graph *PK*_*n*−*ω*,*ω*_, where *PK*_*n*−*ω*,*ω*_ is a graph on *n* vertices obtained from the path *P*_*n*−*ω*_ and the complete graph *K*_*ω*_ by adding an edge between an end vertex of *P*_*n*−*ω*_ and a vertex of *K*_*ω*_ (shown in Figure 1). Furthermore, in this paper, the first four smallest values of the spectral radius are obtained among all connected graphs with maximum clique size *ω*.

Let *I*_*n*,*ω*_ be the set of all connected graphs of order *n* with a maximum clique size *ω*, where 2 ≤ *ω* ≤ *n*. It is easy to see that *I*_*ω*,*ω*_ = {*K*_*ω*_}. By direct calculation, we have *ρ*(*K*_*ω*_) = *ω* − 1. If *G* ∈ *I*_*ω*+1,*ω*_, then, from the Perron-Frobenius theorem, the first *ω* − 1 smallest values of the spectral radius of *I*_*ω*+1,*ω*_ are *PK*_1,*ω*;*i*_ (0 ≤ *i* ≤ *ω* − 2), respectively, where *PK*_1,*ω*;*i*_ is the graph obtained from *PK*_1,*ω*_ by adding *i* (0 ≤ *i* ≤ *ω* − 2) edges. So in the following, we consider that *n* ≥ *ω* + 2.

## 2. Preliminaries

In order to complete the proof of our main result, we need the following lemmas.

**Lemma 1 (see [6]).**

Let *v* be a vertex of the graph *G*. Then the inequalities

(2) λ1(G)≥λ1(G−v)≥λ2(G)≥λ2(G−v)≥⋯≥λn−1(G−v)≥λn(G)

hold. If *G* is connected, then *λ*_1_(*G*) > *λ*_1_(*G* − *v*).

For the spectral radius of a graph, by the well-known Perron-Frobenius theory, we have the following.

**Lemma 2 .**

Let *G* be a connected graph and *H* a proper subgraph of *G*. Then *ρ*(*H*) < *ρ*(*G*).

**Lemma 3 (see [6, 7]).**

Let *G* be a graph on *n* vertices, then

(3) $$\begin{array}{c} \rho \left(G\right)\leq \max\left\{d\left(v\right):v\in V\left(G\right)\right\}. \end{array}$$

The equality holds if and only if *G* is a regular graph.

**Figure 2:**
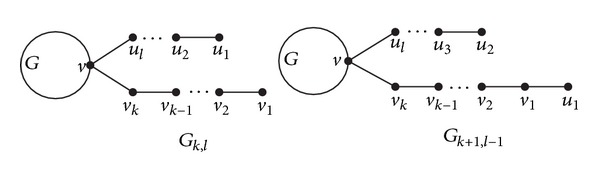
Grafting an edge.

Let *v* be a vertex of a graph *G* and suppose that two new paths *P* = *v*(*v*_*k*+1_)*v*_*k*_ ⋯ *v*_2_ *v*_1_ and *Q* = *v*(*u*_*l*+1_)*u*_*l*_ ⋯ *u*_2_ *u*_1_ of lengths *k* and *l* (*k* ≥ *l* ≥ 1) are attached to *G* at *v*( = *v*_*k*+1_ = *u*_*l*+1_), respectively, to form a new graph *G*_*k*,*l*_ (shown in Figure 2), where *v*_1_, *v*_2_,…, *v*_*k*_ and *u*_1_, *u*_2_,…, *u*_*l*_ are distinct. Let

(4) $$\begin{array}{c} G_{k+1,l-1}=G_{k,l}-u_{1}u_{2}+v_{1}u_{1}. \end{array}$$

We call that *G*_*k*+1,*l*−1_ is obtained from *G*_*k*,*l*_ by grafting an edge (see Figure 2).

**Lemma 4 (see [8, 9]).**

Let *G* be a connected graph on *n* ≥ 2 vertices and *v* is a vertex of *G*. Let *G*_*k*,*l*_ and *G*_*k*+1,*l*−1_ (*k* ≥ *l* ≥ 1) be the graphs as defined above. Then *ρ*(*G*_*k*,*l*_) > *ρ*(*G*_*k*+1,*l*−1_).

Let *v* be a vertex of the graph *G* and *N*(*v*) the set of vertices adjacent to *v*.

**Lemma 5 (see [10, 11]).**

Let *G* be a connected graph, and let *u*, *v* be two vertices of *G*. Suppose that *v*_1_, *v*_2_,…, *v*_*s*_ ∈ *N*(*v*)∖(*N*(*u*)⋃{*u*}) (1 ≤ *s* ≤ *d*(*v*)) and *x* = (*x*_1_, *x*_2_,…, *x*_*n*_) is the Perron vector of *G*, where *x*_*i*_ corresponds to the vertex *v*_*i*_ (1 ≤ *i* ≤ *n*). Let G* be the graph obtained from *G* by deleting the edges *vv*_*i*_ and adding the edges *uv*_*i*_ (1 ≤ *i* ≤ *s*). If *x*_*u*_ ≥ *x*_*v*_, then *ρ*(*G*) < *ρ*(G*).

**Lemma 6 (see [12]).**

Let *v* be a vertex of *G*, let *φ*(*v*) be the collection of circuits containing *v*, and let *V*(*Z*) denote the set of vertices in the circuit *Z*. Then the characteristic polynomial Φ(*G*) satisfies

(5) Φ(G)=xΦ(G−v)−∑wΦ(G−v−w) −2∑Z∈φ(v)Φ(G−V(Z)),

where the first summation extends over those vertices *w* adjacent to *v*, and the second summation extends over all *Z* ∈ *φ*(*v*).

An internal path of a graph *G* is a sequence of vertices *v*_1_, *v*_2_,…, *v*_*k*_ with *k* ≥ 2 such that

1. the vertices in the sequence are distinct (except possibly *v*_1_ = *v*_*k*_);
2. *v*_*i*_ is adjacent to *v*_*i*+1_, (*i* = 1,2,…, *k* − 1);
3. the vertex degrees *d*(*v*_*i*_) satisfy *d*(*v*_1_) ≥ 3, *d*(*v*_2_) = ⋯ = *d*(*v*_*k*−1_) = 2 (unless *k* = 2) and *d*(*v*_*k*_) ≥ 3.

Let *W*_*n*_ be the tree on *n* vertices obtained from *P*_*n*−4_ by attaching two new pendant edges to each end vertex of *P*_*n*−4_, respectively.

**Lemma 7 (see [13]).**

Suppose that *G* ≠ *W*_*n*_ is a connected graph and *uv* is an edge on an internal path of *G*. Let *G*_*uv*_ be the graph obtained from *G* by subdivision of the edge *uv*. Then *ρ*(*G*_*uv*_) < *ρ*(*G*).

## 3. Main Results

**Figure 3:**
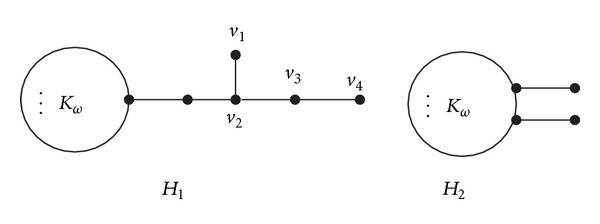
*H*_1_ and *H*_2_.

Let *H*_1_ be the graph obtained from *K*_*ω*_ and a path *P*_4_ : *v*_1_ *v*_2_ *v*_3_ *v*_4_ by joining a vertex of *K*_*ω*_ and a nonpendant vertex, say, *v*_2_, of *P*_4_ by a path with length 2 and let *H*_2_ be the graph obtained from *K*_*ω*_ by attaching two pendant edges at two different vertices of *K*_*ω*_ (see Figure 3).

**Lemma 8 .**

Let *H*_1_ and *H*_2_ be the graphs defined as above (see Figure 3). If *ω* ≥ 3, then *ρ*(*H*_2_) > *ρ*(*H*_1_).

**Proof**

For 5 ≥ *ω* ≥ 3, by direct computations, we have *ρ*(*H*_2_) > *ρ*(*H*_1_). In the following, we suppose that *ω* ≥ 6. From Lemma 6, we have

(6) Φ(H1)=(x+1)ω−2[x7−(ω−2)x6−(ω+4)x5+(5ω−10)x4+(4ω+1)x3−(5ω−10)x2−(2ω−1)x+ω−2]=(x−ω+2)ω−2g1(x).Φ(H2)=(x+1)ω−3[x5−(ω−3)x4−(2ω−1)x3+(ω−5)x2+(2ω−3)x−ω+3]=(x+1)ω−3g2(x).

By direct calculation, we have

(7) g1(ω−1+1ω2) =−ω3+2ω2+6ω+13ω+26ω2−54ω3  +26ω4+34ω5−54ω6+20ω7+20ω8−25ω9  +5ω10+6ω11−5ω12+1ω14−20<0;g1(ω−1+2ω2) =ω4−6ω3+7ω2+26ω+66ω+166ω2−416ω3  +224ω4+432ω5−832ω6+320ω7+560ω8−800ω9  +160ω10+384ω11−320ω12+128ω14−91>0;g2(ω−1+2ω2) =−2ω+12ω−18ω2−8ω3+48ω4−48ω5  −8ω6+64ω7−32ω8+32ω10<0.

From Lemmas 1 and 3, we have *ω* > *ρ*(*H*_1_) ≥ *ρ*(*K*_*ω*_) = *ω* − 1 ≥ *λ*_2_(*H*_1_) and *ω* > *ρ*(*H*_2_) ≥ *ρ*(*K*_*ω*_) = *ω* − 1. Then from (7) we have *ρ*(*H*_2_) > *ω* − 1 + (2/*ω* ^2^) > *ρ*(*H*_1_).

**Figure 4:**
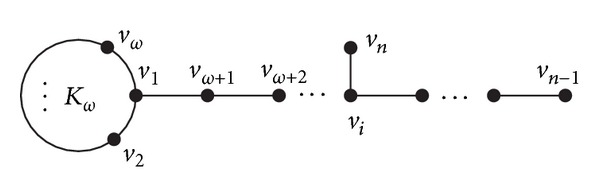
Graph *PK*_*n*−*ω*,*ω*_^*i*^, where *i* = *ω* + 1,…, *n* − 1.

Let *PK*_*n*−*ω*,*ω*_^*i*^ be the graph obtained from the kite graph *PK*_*n*−*ω*−1,*ω*_ (see Figure 1) and an isolated vertex *v*_*n*_ by adding an edge *v*_*n*_ *v*_*i*_ (*ω* + 1 ≤ *i* ≤ *n* − 1) (see Figure 4). It is easy to see that *PK*_5,*ω*_^*ω*+2^ = *H*_1_ and *PK*_*n*−*ω*,*ω*_^*n*−1^ = *PK*_*n*−*ω*,*ω*_.

**Figure 5:**
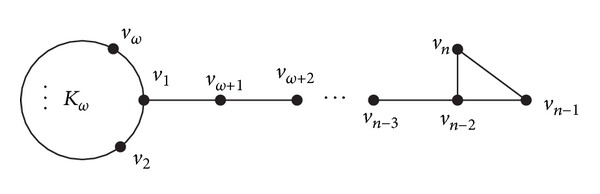
Graph ${\bar{PK}}_{n-\omega ,\omega }^{n-2}$.

Let ${\bar{PK}}_{n-\omega ,\omega }^{n-2}=PK_{n-\omega ,\omega }^{n-2}+v_{n-1}v_{n}$ (see Figure 5).

**Lemma 9 .**

Let *PK*_*n*−*ω*,*ω*_^*i*^ be the graphs defined as above (see Figure 4). Then

(8) ρ(Pn)<ρ(PKn−2,2n−2)<ρ(Cn)=ρ(Wn)<ρ(PKn−2,2n−3),(n≥10).

**Proof**

Clearly, *P*_*n*_ = *P*_2_*n*−2,0__, *PK*_*n*−2,2_^2^ = *P*_2_*n*−3,1__. From Lemma 4, we have

(9) $$\begin{array}{c} \rho \left(P_{n}\right)<\rho \left(PK_{n-2,2}^{n-2}\right)<\rho \left(W_{n}\right)=2=\rho \left(C_{n}\right). \end{array}$$

For *n* ≥ 10, from Lemma 2, we have *ρ*(*PK*_*n*−2,2_^*n*−3^) ≥ *ρ*(*PK*_8,2_^7^) ≈ 2.00659 > *ρ*(*C*_*n*_).

**Figure 6:**
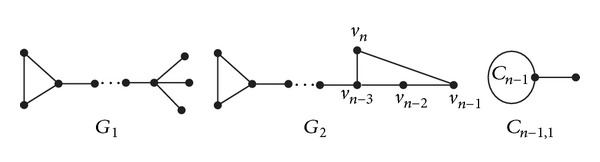
Graphs *G*_1_, *G*_2_, *C*_*n*−1,1_.

Let *G*_1_ = *PK*_*n*−3,3_^*n*−3^ − *v*_*n*−1_ *v*_*n*−2_ + *v*_*n*−3_ *v*_*n*−1_, let *G*_2_ = *PK*_*n*−3,3_^*n*−3^ + *v*_*n*−1_ *v*_*n*_, and let *C*_*n*−1,1_ be the graph obtained from *C*_*n*−1_ and an isolated vertex by adding an edge between some vertex of *C*_*n*−1_ and the isolated vertex (see Figure 6).

**Theorem 10 .**

Among all connected graphs on *n* vertices with maximum clique size *ω* = 2 and *n* ≥ 10, the first four smallest spectral radii are exactly obtained for *P*_*n*_, *PK*_*n*−2,2_^*n*−2^, *C*_*n*_, *W*_*n*_, and *PK*_*n*−2,2_^*n*−3^, respectively.

**Proof**

Let *G* be a connected graph with maximum clique size *ω* = 2 and *n* ≥ 10 vertices. From Lemma 9, we have *ρ*(*P*_*n*_) < *ρ*(*PK*_*n*−2,2_^*n*−2^) < *ρ*(*W*_*n*_) = *ρ*(*C*_*n*_) < *ρ*(*PK*_*n*−2,2_^*n*−3^). Thus, we only need to prove that *ρ*(*G*) > *ρ*(*PK*_*n*−2,2_^*n*−3^) if *G* ≠ *P*_*n*_, *PK*_*n*−2,2_^*n*−2^, *W*_*n*_, *C*_*n*_, *PK*_*n*−2,2_^*n*−3^. If *G* is a tree, note that *G* ≠ *P*_*n*_, *PK*_*n*−2,2_^*n*−2^, *W*_*n*_, *PK*_*n*−2,2_^*n*−3^, then, from Lemma 4, we have *ρ*(*G*) > *ρ*(*PK*_*n*−2,2_^*n*−3^). If *G* contains some cycle as a subgraph, then, from Lemmas 2 and 7, we have *ρ*(*G*) ≥ *ρ*(*C*_*n*−1,1_) > *ρ*(*PK*_*n*−2,2_^*n*−3^).

**Lemma 11 .**

Let *PK*_*n*−*ω*,*ω*_^*i*^, ${\bar{PK}}_{n-\omega ,\omega }^{n-2}$, *G*_1_ and *G*_2_ be the graphs defined as above (see Figures 4, 5, and 6). Then

(10) ρ(PKn−3,3n−4)<min⁡{ρ(PK¯n−3,3n−2),ρ(G1),ρ(G2)},(n≥8).

**Proof**

For 8 ≤ *n* ≤ 11, by direct calculation, we have *ρ*(*PK*_*n*−3,3_^*n*−4^) < *ρ*(*G*_1_). If *n* ≥ 12, from Lemmas 2 and 7, we have 2.23601 < *ρ*(*PK*_8,3_) < *ρ*(*PK*_*n*−3,3_^*n*−4^) < *ρ*(*PK*_9,3_^8^) < 2.23808. From Lemma 6, we have

(11) Φ(PKn−3,3n−4) =(x5−4x3+3x)Φ(PKn−8,3)  −(x4−2x2)Φ(PKn−8,3−vn−5) =f1(x)Φ(PKn−8,3)−f2(x)Φ(PKn−8,3−vn−5),Φ(G1)=(x5−4x3)Φ(PKn−8,3) −(x4−3x2)Φ(PKn−8,3−vn−5)=f3(x)Φ(PKn−8,3)−f4(x)Φ(PKn−8,3−vn−5).

Then we have

(12) f3(x)Φ(PKn−3,3n−4)−f1(x)Φ(G1) =(f1(x)f4(x)−f2(x)f3(x))Φ(PKn−8,3−vn−5) =(−x7+7x5−9x3)Φ(PKn−8,3−vn−5) =R1(x)Φ(PKn−8,3−vn−5).

For 2.23601 < *x* < 2.23808, we have

(13) f1(x)>2.236015−4×2.238083+3 ×2.23601≈17>0;f3(x)>2.236015−4×2.238083≈11>0;R1(x)>−2.238087+7×2.236015 −9×2.238083≈9>0.

Note that from Lemma 2, *ρ*(*PK*_*n*−8,3_ − *v*_*n*−5_) < *ρ*(*PK*_*n*−3,3_^*n*−4^) and 2.23601 < *ρ*(*PK*_*n*−3,3_^*n*−4^) < 2.23808. Then, we have

(14) f3(x)Φ(PKn−3,3n−4)>f1(x)Φ(G1),x∈[ρ(PKn−3,3n−4),2.23808).

Thus, *ρ*(*PK*_*n*−3,3_^*n*−4^) < *ρ*(*G*_1_). By similar method, we have for *n* ≥ 8

(15) $$\begin{array}{c} \rho \left(PK_{n-3,3}^{n-4}\right)<\rho \left({\bar{PK}}_{n-3,3}^{n-2}\right),\;\;\rho \left(PK_{n-3,3}^{n-4}\right)<\rho \left(G_{2}\right). \end{array}$$

**Figure 7:**
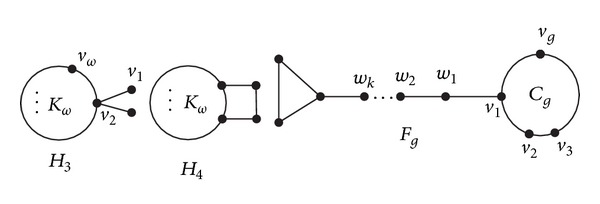
Graphs *H*_3_, *H*_4_, and *F*_*g*_.

Let *H*_3_ be the graph obtained from *K*_*ω*_ by attaching two pendant edges at some vertex of *K*_*ω*_; let *H*_4_ be the graph obtained from *K*_*ω*_ and *P*_2_ by adding two edges between two vertices of *K*_*ω*_ and two end vertices of *P*_2_ (see Figure 7).

**Theorem 12 .**

Among all connected graphs on *n* vertices with maximum clique size *ω* = 3 and *n* ≥ 9, the first four smallest spectral radii are exactly obtained for *PK*_*n*−3,3_, *PK*_*n*−3,3_^*n*−2^, *PK*_*n*−3,3_^*n*−3^, *PK*_*n*−3,3_^*n*−4^, respectively.

**Proof**

Let *G* be a connected graph with maximum clique size *ω* = 3 and *n* ≥ 9 vertices. From Lemmas 2 and 7, we have

(16) $$\begin{array}{c} \rho \left(PK_{n-3,3}^{n-4}\right)>\rho \left(PK_{n-3,3}^{n-3}\right)>\rho \left(PK_{n-3,3}^{n-2}\right)>\rho \left(PK_{n-3,3}\right). \end{array}$$

Thus, we only need to prove that *ρ*(*G*) > *ρ*(*PK*_*n*−3,3_^*n*−4^) if *G* ≠ *PK*_*n*−3,3_, *PK*_*n*−3,3_^*n*−2^, *PK*_*n*−3,3_^*n*−3^, *PK*_*n*−3,3_^*n*−4^.

We distinguish the following three cases.

*Case  1.* If there exist at least two vertices outside of *K*_3_ that are adjacent to some vertices of *K*_3_, then we have that *G* contains either *H*_2_ (*ω* = 3) or *H*_3_ (*ω* = 3) as a proper subgraph. If *G* contains *H*_2_ (*ω* = 3) as a proper subgraph, from Lemmas 2 and 7, we have

(17) ρ(G)>ρ(H2)≈2.30278>ρ(PK6,35)≈2.26542>ρ(PKn−3,3n−4), (ω=3).

If *G* contains *H*_3_ (*ω* = 3) as a proper subgraph, from Lemmas 2 and 7, we have

(18) ρ(G)>ρ(H3)≈2.34292>ρ(PK6,35)>ρ(PKn−3,3n−4), (ω=3).

*Case  2.* Suppose that there exists a vertex, say, *u*, which does not belong to *K*_3_, such that *u* is adjacent to at least two vertices of *K*_3_. Then *G* contains *C*_4_* as a proper subgraph, where *C*_4_* is obtained from *C*_4_ by adding an edge between two disjoint vertices. From Lemmas 2 and 7, we have

(19) $$\begin{array}{c} \rho \left(G\right)>\rho \left(C_{4}^{*}\right)\approx 2.56155>\rho \left(PK_{6,3}^{5}\right)>\rho \left(PK_{n-3,3}^{n-4}\right). \end{array}$$

*Case  3.* Suppose that there uniquely exists a vertex *u* which does not belong to *K*_3_ such that *u* is adjacent to a vertex of *K*_3_. We distinguish the following two cases.

*Subcase 1.* Suppose that *G* − *V*(*K*_3_) is a tree. If there exist two vertices *u*, *r* ∈ *V*(*G* − *V*(*K*_3_)) such that *d*(*u*) ≥ 3 and *d*(*r*) ≥ 3, then, from Lemmas 2, 4, and 7, we have *ρ*(*G*) > *ρ*(*PK*_*n*−3,3_^*n*−4^). If there exists only one vertex *u* ∈ *V*(*G* − *V*(*K*_3_)) such that *d*(*u*) ≥ 4, then, from Lemmas 2, 7, and 11, we have *ρ*(*G*) ≥ *ρ*(*G*_1_) > *ρ*(*PK*_*n*−3,3_^*n*−4^). If there exists exactly one vertex *u* ∈ *V*(*G* − *V*(*K*_3_)) such that *d*(*u*) = 3, note that *G* ≠ *PK*_*n*−3,3_^*n*−2^, *PK*_*n*−3,3_^*n*−3^, *PK*_*n*−3,3_^*n*−4^, then from Lemmas 2 and 7 we have *ρ*(*G*) > *ρ*(*PK*_*n*−3,3_^*n*−4^).

*Subcase 2.* Suppose that *G* − *V*(*K*_3_) contains cycle *C*_*g*_ as a subgraph. If *g* = 3,4, then, from Lemmas 2, 7 and 11, we have $\rho (G)\geq \rho ({\bar{PK}}_{n-3,3}^{n-2})>\rho (PK_{n-3,3}^{n-4})$ or *ρ*(*G*) ≥ *ρ*(*G*_2_) > *ρ*(*PK*_*n*−3,3_^*n*−4^). If *g* ≥ 5, then, from Lemma 2, we can construct a graph *F*_*g*_ from *G* by deleting vertices such that *ρ*(*G*) ≥ *ρ*(*F*_*g*_), where *F*_*g*_ is the graph obtained from *K*_3_ and a cycle *C*_*g*_ by joining a vertex of *K*_3_ and a vertex of *C*_*g*_ with a path and |*V*(*F*_*g*_)|≤*n* (see Figure 7). Suppose that *C*_*g*_ is labelled *v*_1_, *v*_2_,…, *v*_*g*_ satisfying *v*_*i*_ *v*_*i*+1_ ∈ *E*(*C*_*g*_), (1 ≤ *i* ≤ *g* − 1), *v*_1_ *v*_*g*_ ∈ *E*(*C*_*g*_), and *d*(*v*_1_) = 3. Then, from Lemmas 2 and 7, we have *ρ*(*F*_*g*_ − *v*_2_ *v*_3_) > *ρ*(*PK*_*n*−3,3_^*n*−4^). Thus, we have *ρ*(*G*) > *ρ*(*PK*_*n*−3,3_^*n*−4^).

**Lemma 13 .**

Let *PK*_*n*−*ω*,*ω*_^*i*^ and ${\bar{PK}}_{n-\omega ,\omega }^{n-2}$ be the graphs defined as above (see Figures 4 and 5). Then $\rho (PK_{n-\omega ,\omega }^{n-3})>\rho ({\bar{PK}}_{n-\omega ,\omega }^{n-2})$ (*ω* ≥ 4).

**Proof**

Let *X* = (*x*_1_, *x*_2_,…, *x*_*n*_)^*T*^ be the Perron vector of ${\bar{PK}}_{n-\omega ,\omega }^{n-2}$, where *x*_*i*_ corresponds to *v*_*i*_. It is easy to prove that *x*_*n*_ = *x*_*n*−1_. From $AX=\rho ({\bar{PK}}_{n-\omega ,\omega }^{n-2})X$, we have

(20) xn−2=(ρ(PK¯n−ω,ωn−2)−1)xn,xn−3=[ρ(PK¯n−ω,ωn−2)(ρ(PK¯n−ω,ωn−2)−1)−2]xn.

From Lemma 2, for *ω* ≥ 4 we have $\rho ({\bar{PK}}_{n-\omega ,\omega }^{n-2})\geq \rho (K_{\omega })=\omega -1\geq 3$. Then

(21) ρ(PKn−ω,ωn−3)−ρ(PK¯n−ω,ωn−2) ≥XTA(PKn−ω,ωn−3)X−XTA(PK¯n−ω,ωn−2)X =2xn(xn−3−xn−2−xn) =2[ρ(PK¯n−ω,ωn−2)(ρ(PK¯n−ω,ωn−2)−2)−2]xn ≥2xn>0.

So, $\rho (PK_{n-\omega ,\omega }^{n-3})>\rho ({\bar{PK}}_{n-\omega ,\omega }^{n-2})$.

**Figure 8:**
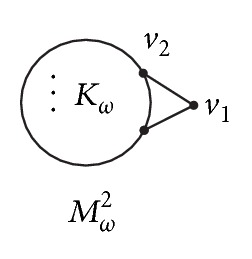
Graph *M*_*ω*_^2^.

Let *M*_*ω*_^2^ (*ω* ≥ 4) be the graph as shown in Figure 8.

**Theorem 14 .**

Among all connected graphs on *n* vertices with maximum clique size *ω* ≥ 4 and *n* ≥ *ω* + 5, the first four smallest spectral radii are exactly obtained for *PK*_*n*−*ω*,*ω*_, *PK*_*n*−*ω*,*ω*_^*n*−2^, ${\bar{PK}}_{n-\omega ,\omega }^{n-2}$, *PK*_*n*−*ω*,*ω*_^*n*−3^, respectively.

**Proof**

Let *G* be a connected graph with maximum clique size *ω* ≥ 4 and *n* ≥ *ω* + 5 vertices. Suppose that *K*_*ω*_ is a maximum clique of *G*. From Lemmas 2, 4, and 13, we have

(22) $$\begin{array}{c} \rho \left(PK_{n-\omega ,\omega }^{n-3}\right)>\rho \left({\bar{PK}}_{n-\omega ,\omega }^{n-2}\right)>\rho \left(PK_{n-\omega ,\omega }^{n-2}\right)>\rho \left(PK_{n-\omega ,\omega }\right). \end{array}$$

Thus, we only need to prove that *ρ*(*G*) > *ρ*(*PK*_*n*−*ω*,*ω*_^*n*−3^) if *G* ≠ *PK*_*n*−*ω*,*ω*_, *PK*_*n*−*ω*,*ω*_^*n*−2^, ${\bar{PK}}_{n-\omega ,\omega }^{n-2}$, *PK*_*n*−*ω*,*ω*_^*n*−3^. We distinguish the following three cases.

*Case  1.* If there exist at least two vertices outside of *K*_*ω*_ that are adjacent to some vertices of *K*_*ω*_, then *G* contains either *H*_2_ or *H*_3_ as a proper subgraph. If *G* contains *H*_2_ as a proper subgraph, from Lemmas 2, 7, and 8, we have

(23) $$\begin{array}{c} \rho \left(G\right)>\rho \left(H_{2}\right)>\rho \left(H_{1}\right)\geq \rho \left(PK_{n-\omega ,\omega }^{n-3}\right). \end{array}$$

If *G* contains *H*_3_ as a proper subgraph, from Lemmas 2, 5, 7, and 8, we have

(24) $$\begin{array}{c} \rho \left(G\right)>\rho \left(H_{3}\right)>\rho \left(H_{2}\right)>\rho \left(H_{1}\right)\geq \rho \left(PK_{n-\omega ,\omega }^{n-3}\right). \end{array}$$

*Case  2.* Suppose that there exists a vertex, say, *u*, which does not belong to *K*_*ω*_, such that *u* is adjacent to at least two vertices of *K*_*ω*_. From Lemmas 2, 7, and 8, we have

(25) $$\begin{array}{c} \rho \left(G\right)>\rho \left(M_{\omega }^{2}\right)>\rho \left(H_{4}\right)>\rho \left(H_{2}\right)>\rho \left(H_{1}\right)\geq \rho \left(PK_{n-\omega ,\omega }^{n-3}\right). \end{array}$$

*Case  3.* Suppose that there uniquely exists a vertex *u* which does not belong to *K*_*ω*_ such that *u* is adjacent to a vertex of *K*_*ω*_. If *G* − *V*(*K*_*ω*_) is a tree, note that *G* ≠ *PK*_*n*−*ω*,*ω*_, *PK*_*n*−*ω*,*ω*_^*n*−2^, *PK*_*n*−*ω*,*ω*_^*n*−3^, then, from Lemmas 2, 4, and 7, we have *ρ*(*G*) > *ρ*(*PK*_*n*−*ω*,*ω*_^*n*−3^). Suppose that *G* − *V*(*K*_*ω*_) contains cycle *C*_*g*_ as a subgraph. If *g* = 3, note that $G\neq {\bar{PK}}_{n-\omega ,\omega }^{n-2}$, then, from Lemmas 2 and 7, we have *ρ*(*G*) > *ρ*(G*) > *ρ*(*PK*_*n*−*ω*,*ω*_^*n*−3^), where G* = *PK*_*n*−*ω*,*ω*_^*n*−3^ + *v*_*n*−1_ *v*_*n*_. If *g* ≥ 4, then by the similar reasoning as that of Subcase 2 of Case 3 of Theorem 12, we have *ρ*(*G*) > *ρ*(*PK*_*n*−*ω*,*ω*_^*n*−3^).

**Lemma 15 .**

Let *H*_3_ and *H*_4_ be the graphs defined as above (see Figure 7). Then

(26) $$\begin{array}{c} \rho \left(H_{4}\right)>\rho \left(H_{3}\right)\;\left(\omega \geq 3\right). \end{array}$$

**Proof**

Let *X* = (*x*_1_, *x*_2_,…, *x*_*n*_)^*T*^ be the Perron vector of *H*_3_, where *x*_*i*_ corresponds to *v*_*i*_. From *AX* = *ρ*(*H*_3_)*X*, we have

(27) $$\begin{array}{c} \rho \left(H_{3}\right)x_{1}=x_{2},\\ \rho \left(H_{3}\right)x_{2}=2x_{1}+\left(\omega -1\right)x_{\omega },\\ \rho \left(H_{3}\right)x_{\omega }=\left(\omega -2\right)x_{\omega }+x_{2}. \end{array}$$

From above equations, we have

(28) $$\begin{array}{c} \rho^{3}\left(H_{3}\right)-\left(\omega -2\right)\rho^{2}\left(H_{3}\right)-\left(\omega +1\right)\rho \left(H_{3}\right)+2\omega -4=0. \end{array}$$

Let

(29) $$\begin{array}{c} r_{1}\left(x\right)=x^{3}-\left(\omega -2\right)x^{2}-\left(\omega +1\right)x+2\omega -4. \end{array}$$

Then

(30) $$\begin{array}{c} r_{1}\left(\omega -1\right)=-2<0. \end{array}$$

For *x* > *ω* − 1 and *ω* ≥ 3, we have

(31) $$\begin{array}{c} r_{1}^{\prime }\left(x\right)=3x^{2}-2\left(\omega -2\right)x-\left(\omega +1\right)>0. \end{array}$$

Note that *ρ*(*H*_3_) > *ρ*(*K*_*ω*_) = *ω* − 1. From (30) and (31), we have *ρ*(*H*_3_) which is the largest root of equation *r*_1_(*x*) = 0. Similarly, we have *ρ*(*H*_4_) which is the largest root of equation

(32) $$\begin{array}{c} r_{2}\left(x\right)=x^{3}-\left(\omega -1\right)x^{2}-2x+2\omega -4=0. \end{array}$$

Then we have, for *x* > *ω* − 1,

(33) $$\begin{array}{c} r_{1}\left(x\right)-r_{2}\left(x\right)=x^{2}-\left(\omega -1\right)x>0. \end{array}$$

Thus, we have *ρ*(*H*_3_) < *ρ*(*H*_4_).

**Theorem 16 .**

Let *G* be a graph on *n* vertices with maximum clique size *ω* ≥ 3 and *n* = *ω* + 2. Let *PK*_2,*ω*_, *H*_2_, *H*_3_, and *H*_4_ be the graphs defined as above (see Figures 1, 3 and 7). The first four smallest spectral radii are obtained for *PK*_2,*ω*_, *H*_2_, *H*_3_, *H*_4_, respectively.

**Proof**

From Lemmas 2, 5, 8, and 15, we have

(34) $$\begin{array}{c} \rho \left(H_{4}\right)>\rho \left(H_{3}\right)>\rho \left(H_{2}\right)>\rho \left(H_{1}\right)>\rho \left(PK_{2,\omega }\right). \end{array}$$

Thus, we only need to prove that, for *G* ≠ *PK*_2,*ω*_, *H*_2_, *H*_3_, and *H*_4_, *ρ*(*G*) > *ρ*(*H*_4_). We distinguish the following two cases.

*Case  1.* Suppose that there exists exactly one vertex outside of *K*_*ω*_ that is adjacent to at least two vertices of *K*_*ω*_. Then *G* contains *M*_*ω*_^2^ (see Figure 8) as a subgraph. From Lemmas 2 and 7, we have *ρ*(*M*_*ω*_^2^) > *ρ*(*H*_4_).

*Case  2.* Suppose that the two vertices outside of *K*_*ω*_ that are all adjacent to some vertices of *K*_*ω*_. Note that *G* ≠ *H*_2_, *H*_3_, *H*_4_. Then *G* contains one of graphs ${\bar{H}}_{3}$ and *M*_*ω*_^2^ as a subgraph, where ${\bar{H}}_{3}$ is obtained from *H*_3_ by adding an edge between two pendant vertices. From Lemma 5, we have $\rho (G)\geq \rho ({\bar{H}}_{3})>\rho (H_{4})$. From Lemmas 2 and 7, *ρ*(*G*) > *ρ*(*M*_*ω*_^2^) > *ρ*(*H*_4_).

**Figure 9:**
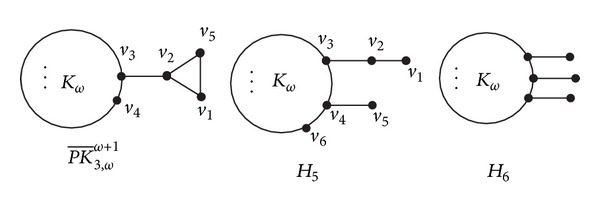
Graphs ${\bar{PK}}_{3,\omega }^{\omega +1}$, *H*_5_, and *H*_6_.

Let *H*_5_ be the graph obtained from *H*_2_ and an isolated vertex by adding an edge between a pendant vertex of *H*_2_ and the isolated vertex; let ${\bar{PK}}_{3,\omega }^{\omega +1}$ and *H*_6_ be the graphs as shown in Figure 9.

**Lemma 17 .**

Let ${\bar{PK}}_{3,\omega }^{\omega +1}$ and *H*_5_ be the graphs defined as above (see Figure 9). Then

(35) $$\begin{array}{c} \rho \left(H_{5}\right)>\rho \left({\bar{PK}}_{3,\omega }^{\omega +1}\right),\;\left(\omega \geq 4\right). \end{array}$$

**Proof**

Let *X* = (*x*_1_, *x*_2_,…, *x*_*n*_)^*T*^ be the Perron vector of ${\bar{PK}}_{3,\omega }^{\omega +1}$, where *x*_*i*_ corresponds to *v*_*i*_. It is easy to see that *x*_1_ = *x*_5_. From $AX=\rho ({\bar{PK}}_{3,\omega }^{\omega +1})X$, we have

(36) $$\begin{array}{c} \rho \left({\bar{PK}}_{3,\omega }^{\omega +1}\right)x_{1}=x_{1}+x_{2},\\ \rho \left({\bar{PK}}_{3,\omega }^{\omega +1}\right)x_{2}=2x_{1}+x_{3},\\ \rho \left({\bar{PK}}_{3,\omega }^{\omega +1}\right)x_{3}=x_{2}+\left(\omega -1\right)x_{4},\\ \rho \left({\bar{PK}}_{3,\omega }^{\omega +1}\right)x_{4}=x_{3}+\left(\omega -2\right)x_{4}. \end{array}$$

From above equations, we have

(37) $$\begin{array}{c} x_{2}=\left(\rho \left({\bar{PK}}_{3,\omega }^{\omega +1}\right)-1\right)x_{1},\\ x_{4}=\frac{\rho^{2}\left({\bar{PK}}_{3,\omega }^{\omega +1}\right)-\rho \left({\bar{PK}}_{3,\omega }^{\omega +1}\right)-2}{\rho \left({\bar{PK}}_{3,\omega }^{\omega +1}\right)-\omega +2}x_{1}. \end{array}$$

Then for *ω* ≥ 4, we have

(38) ρ(H5)−ρ(PK¯3,ωω+1)≥XTA(H5)X−XTA(PK¯3,ωω+1)X=2x1(x4−x2−x1)=2(ω−3)ρ(PK¯3,ωω+1)−2ρ(PK¯3,ωω+1)−ω+2x1>0.

The result follows.

**Lemma 18 .**

Let *H*_5_ and *H*_6_ be the graphs defined as above (see Figure 9). Then

(39) $$\begin{array}{c} \rho \left(H_{6}\right)>\rho \left(H_{5}\right),\;\left(\omega \geq 4\right). \end{array}$$

**Proof**

For *ω* = 4, by direct calculation, we have *ρ*(*H*_6_) > *ρ*(*H*_5_). In the following, we suppose that *ω* ≥ 5. Then, from Lemmas 2 and 3, we have *ω* > *ρ*(*H*_5_) > *ρ*(*K*_*ω*_) = *ω* − 1 ≥ 4. Let *X* = (*x*_1_, *x*_2_,…, *x*_*n*_)^*T*^ be the Perron vector of *H*_5_, where *x*_*i*_ corresponds to *v*_*i*_. From *AX* = *ρ*(*H*_5_)*X*, we have

(40) $$\begin{array}{c} \rho \left(H_{5}\right)x_{1}=x_{2},\\ \rho \left(H_{5}\right)x_{2}=x_{1}+x_{3},\\ \rho \left(H_{5}\right)x_{3}=x_{2}+x_{4}+\left(\omega -2\right)x_{6},\\ \rho \left(H_{5}\right)x_{4}=x_{3}+x_{5}+\left(\omega -2\right)x_{6},\\ \rho \left(H_{5}\right)x_{5}=x_{4},\\ \rho \left(H_{5}\right)x_{6}=x_{3}+x_{4}+\left(\omega -3\right)x_{6}. \end{array}$$

From above equations, we have for *ω* > *ρ*(*H*_5_) > *ω* − 1 ≥ 4,

(41) x6=ρ2(H5)−1ρ(H5)−ω+3x1 +(ρ2(H5)+ρ(H5))(ρ2(H5)−1)−ρ2(H5)(ρ(H5)−ω+3)(ρ2(H5)+ρ(H5)−1)x1>ρ2(H5)−13x1>ρ(H5)x1=x2.

Then, from Lemma 5, we have *ρ*(*H*_6_) = *ρ*(*H*_5_ − *v*_1_ *v*_2_ + *v*_1_ *v*_6_) > *ρ*(*H*_5_).

**Figure 10:**
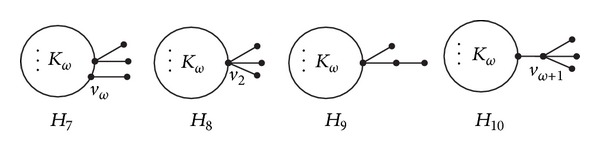
Graphs *H*_7_, *H*_8_, *H*_9_, *H*_10_.

Let *H*_7_ be the graph obtained from *H*_3_ and an isolated vertex by adding an edge between *v*_*ω*_ and the isolated vertex; let *H*_8_ be the graph obtained from *H*_3_ and an isolated vertex by adding an edge between *v*_2_ and the isolated vertex; let *H*_9_ be the graph obtained from *H*_3_ and an isolated vertex by adding an edge between one pendant vertex and the isolated vertex; and let *H*_10_ be the graph obtained from *PK*_3,*ω*_^*ω*+1^ and an isolated vertex by adding an edge between *v*_*ω*+1_ and the isolated vertex (see Figure 10).

**Theorem 19 .**

Let *PK*_3,*ω*_, *PK*_3,*ω*_^*ω*+1^, ${\bar{PK}}_{3,\omega }^{\omega +1}$, and *H*_5_ be the graphs defined as above (see Figures 1, 4, 5, and 9). Among all connected graphs on *n* vertices with maximum clique size *ω* and *n* = *ω* + 3 (*ω* ≥ 4), the first four smallest spectral radii are obtained for *PK*_3,*ω*_, *PK*_3,*ω*_^*ω*+1^, ${\bar{PK}}_{3,\omega }^{\omega +1}$, and *H*_5_, respectively.

**Proof**

From Lemmas 2, 4, and 17, we have

(42) $$\begin{array}{c} \rho \left(H_{5}\right)>\rho \left({\bar{PK}}_{3,\omega }^{\omega +1}\right)>\rho \left(PK_{3,\omega }^{\omega +1}\right)>\rho \left(PK_{3,\omega }\right). \end{array}$$

Thus, we only need to prove that *ρ*(*G*) > *ρ*(*H*_5_) if *G* ≠ *PK*_3,*ω*_, *PK*_3,*ω*_^*ω*+1^, ${\bar{PK}}_{3,\omega }^{\omega +1}$, and *H*_5_. We distinguish the following four cases.

*Case  1.* There exists exactly one vertex outside of *K*_*ω*_ that is adjacent to only one vertex of *K*_*ω*_. Then *G* must be one of graphs *PK*_3,*ω*_, *PK*_3,*ω*_^*ω*+1^, and ${\bar{PK}}_{3,\omega }^{\omega +1}$.

*Case  2.* There exists one vertex outside of *K*_*ω*_ that is adjacent to at least two vertices of *K*_*ω*_. Then *G* contains *M*_*ω*_^2^ (see Figure 8) as a proper subgraph. From Lemmas 2 and 7, we have *ρ*(*G*) > *ρ*(*M*_*ω*_^2^) > *ρ*(*H*_5_).

*Case  3.* If there exactly exist two vertices outside of *K*_*ω*_ that are adjacent to some vertices of *K*_*ω*_, then *G* contains *H*_5_ or *H*_9_ (see Figures 9 and 10) as a subgraph. If *G* contains *H*_9_ as a subgraph, then, from Lemmas 2 and 5, we have *ρ*(*G*) ≥ *ρ*(*H*_9_) > *ρ*(*H*_5_). If *G* contains *H*_5_ as a subgraph, note that *G* ≠ *H*_5_, then, from Lemma 2, we have *ρ*(*G*) > *ρ*(*H*_5_).

*Case  4.* If there exist three vertices outside of *K*_*ω*_ that are adjacent to some vertices of *K*_*ω*_, then *G* contains one of graphs *H*_6_, *H*_7_, and *H*_8_ (see Figures 9 and 10) as a subgraph. From Lemmas 5 and 18, we have *ρ*(*H*_8_) > *ρ*(*H*_7_) > *ρ*(*H*_6_) > *ρ*(*H*_5_). Then, from Lemma 2, we have *ρ*(*G*) > *ρ*(*H*_5_).

**Lemma 20 .**

Let *PK*_*n*−*ω*,*ω*_^*i*^ and ${\bar{PK}}_{4,\omega }^{\omega +2}$ be the graphs defined as above (see Figures 4 and 5). Then

(43) $$\begin{array}{c} \rho \left(PK_{4,\omega }^{\omega +1}\right)>\rho \left({\bar{PK}}_{4,\omega }^{\omega +2}\right),\;\left(\omega \geq 4\right). \end{array}$$

**Proof**

From Lemma 6, we have

(44) Φ(PK¯4,ωω+2)=(x4−4x2−2x+1)Φ(Kω) −(x3−3x−2)Φ(Kω−1)=f5(x)Φ(Kω)−f6(x)Φ(Kω−1);Φ(PK4,ωω+1)=(x4−3x2+1)Φ(Kω) −(x3−x)Φ(Kω−1)=f7(x)Φ(Kω)−f8(x)Φ(Kω−1).

Then, we have

(45) f7(x)Φ(PK¯4,ωω+2)−f5(x)Φ(PK4,ωω+1) =(f5(x)f8(x)−f6(x)f7(x))Φ(Kω−1) =(x5−5x3−4x2+2x+2)Φ(Kω−1) =R2(x)Φ(Kω−1).

For *x* > *ω* − 1 (*ω* ≥ 4), we have

(46) $$\begin{array}{c} f_{5}\left(x\right)>0,\;\;f_{7}\left(x\right)>0,\\ R_{2}\left(x\right)>0,\;\;\;\Phi \left(K_{\omega -1}\right)>0. \end{array}$$

From Lemma 2, we have $\rho ({\bar{PK}}_{4,\omega }^{\omega +2})>\rho (K_{\omega })=\omega -1$ and *ρ*(*PK*_4,*ω*_^*ω*+1^) > *ρ*(*K*_*ω*_) = *ω* − 1. Thus, for *x* > *ω* − 1 (*ω* ≥ 4), we have $f_{7}(x)\Phi ({\bar{PK}}_{4,\omega }^{\omega +2})-f_{5}(x)\Phi (PK_{4,\omega }^{\omega +1})>0$. Then $\rho (PK_{4,\omega }^{\omega +1})>\rho ({\bar{PK}}_{4,\omega }^{\omega +2})$, (*ω* ≥ 4).

**Lemma 21 .**

Let *PK*_*n*−*ω*,*ω*_^*i*^ and *H*_2_ be the graphs defined as above (see Figures 3 and 4). Then

(47) $$\begin{array}{c} \rho \left(H_{2}\right)>\rho \left(PK_{4,\omega }^{\omega +1}\right),\;\left(\omega \geq 3\right). \end{array}$$

**Proof**

For *ω* = 3,4, 5, by direct calculation, we have *ρ*(*H*_2_) > *ρ*(*PK*_4,*ω*_^*ω*+1^). In the following, we suppose that *ω* ≥ 6. From Lemma 6, we have

(48) Φ(PK4,ωω+1)=(x+1)ω−2[x6−(ω−2)x5−(ω+3)x4+(4ω−8)x3+(3ω−1)x2−(2ω−4)x−ω+1]=(x+1)ω−2g3(x).

For *ω* ≥ 6, we have

(49) g3(ω−1+1ω2) =−ω2−13ω+4ω2+17ω3−24ω4+6ω5+14ω6  −16ω7+2ω8+5ω9−4ω10+1ω12+7<0;g3(ω−1+2ω2) =ω3−5ω2+2ω−58ω+20ω2+108ω3−192ω4+48ω5  +192ω6−256ω7+32ω8+160ω9−128ω10+64ω12+24>0.

From Lemmas 1 and 3, we have *ω* > *ρ*(*PK*_4,*ω*_^*ω*+1^) ≥ *ρ*(*K*_*ω*_) = *ω* − 1 ≥ *λ*_2_(*PK*_4,*ω*_^*ω*+1^). Then from (49) we have *ω* − 1 + 2/*ω* ^2^ > *ρ*(*PK*_4,*ω*_^*ω*+1^) > *ω* − 1 + 1/*ω* ^2^. From the proof of Lemma 8, we have *ρ*(*H*_2_) > *ω* − 1 + 2/*ω* ^2^ (*ω* ≥ 6). The result follows.

**Theorem 22 .**

Among all connected graphs on *n* vertices with maximum clique size *ω* and *n* = *ω* + 4 (*ω* ≥ 4), the first four smallest spectral radii are obtained for *PK*_4,*ω*_, *PK*_4,*ω*_^*ω*+2^, ${\bar{PK}}_{4,\omega }^{\omega +2}$, and *PK*_4,*ω*_^*ω*+1^ (see Figures 1, 4, and 5), respectively.

**Proof**

Let *G* be a connected graph with maximum clique size *ω* ≥ 4 and *n* = *ω* + 4 vertices. Suppose that *K*_*ω*_ is a maximum clique of *G*. From Lemmas 2, 4, and 20, we have

(50) $$\begin{array}{c} \rho \left(PK_{4,\omega }^{\omega +1}\right)>\rho \left({\bar{PK}}_{4,\omega }^{\omega +2}\right)>\rho \left(PK_{4,\omega }^{\omega +2}\right)>\rho \left(PK_{4,\omega }\right). \end{array}$$

Thus, we only need to prove that *ρ*(*G*) > *ρ*(*PK*_4,*ω*_^*ω*+1^) if *G* ≠ *PK*_4,*ω*_, *PK*_4,*ω*_^*ω*+2^, ${\bar{PK}}_{4,\omega }^{\omega +2}$, *PK*_4,*ω*_^*ω*+1^. We distinguish the following three cases.

*Case  1.* There exists exactly one vertex outside of *K*_*ω*_ that is adjacent to one vertex of *K*_*ω*_.

*Subcase 1.* Suppose that *G* − *V*(*K*_*ω*_) is a tree. If *G* contains exactly one pendant vertex, then *G* = *PK*_4,*ω*_. If *G* contains exactly two pendant vertices, then *G* = *PK*_4,*ω*_^*ω*+1^ or *G* = *PK*_4,*ω*_^*ω*+2^. If *G* contains three pendant vertices, then *G* = *H*_10_ (see Figure 10). From Lemma 4, we have *ρ*(*H*_10_) > *ρ*(*PK*_4,*ω*_^*ω*+1^).

*Subcase 2.* Suppose that *G* − *V*(*K*_*ω*_) contains a cycle. If *G* − *V*(*K*_*ω*_) contains *C*_4_, then *G* contains *H*_11_ as a subgraph, where *H*_11_ is obtained from *PK*_4,*ω*_^*ω*+1^ by adding an edge between two pendant vertices. From Lemma 2, we have *ρ*(*H*_11_) > *ρ*(*PK*_4,*ω*_^*ω*+1^). If *G* − *V*(*K*_*ω*_) does not contain *C*_4_, then $G={\bar{PK}}_{4,\omega }^{\omega +2}$ or *G* contains ${\bar{PK}}_{3,\omega }^{\omega +1}$ as a proper subgraph. From Lemmas 2 and 7, we have $\rho ({\bar{PK}}_{3,\omega }^{\omega +1})>\rho (H_{11})>\rho (PK_{4,\omega }^{\omega +1})$. Note that $G\neq {\bar{PK}}_{4,\omega }^{\omega +2}$. Thus, we have *ρ*(*G*) > *ρ*(*PK*_4,*ω*_^*ω*+1^).

*Case  2.* There exists at least one vertex outside of *K*_*ω*_ that is adjacent to at least two vertices of *K*_*ω*_. Then *G* contains *M*_*ω*_^2^ (see Figure 8) as a subgraph. From Lemmas 2, 7, and 21, we have *ρ*(*G*) > *ρ*(*M*_*ω*_^2^) > *ρ*(*H*_2_) > *ρ*(*PK*_4,*ω*_^*ω*+1^).

*Case  3.* There exist at least two vertices outside of *K*_*ω*_ that are adjacent to some vertices of *K*_*ω*_. Then *G* contains *H*_2_ or *H*_3_ as a subgraph (see Figures 3 and 7). From Lemmas 2, 5, and 21, we have *ρ*(*H*_3_) > *ρ*(*H*_2_) > *ρ*(*PK*_4,*ω*_^*ω*+1^). Thus, from Lemma 2, we have *ρ*(*G*) > *ρ*(*PK*_4,*ω*_^*ω*+1^).

## 4. Conclusion 

In this paper, the first four graphs, which have the smallest values of the spectral radius among all connected graphs of order *n* with maximum clique size *ω* ≥ 2, are determined.
